# Supplementary figures and images for: Stranded short nascent strand sequencing reveals the topology of DNA replication origins in Trypanosoma brucei
Source: eLife. 2026 May 15;14:RP108143. doi: 10.7554/eLife.108143 (PMC13179062; doi:10.7554/eLife.108143)

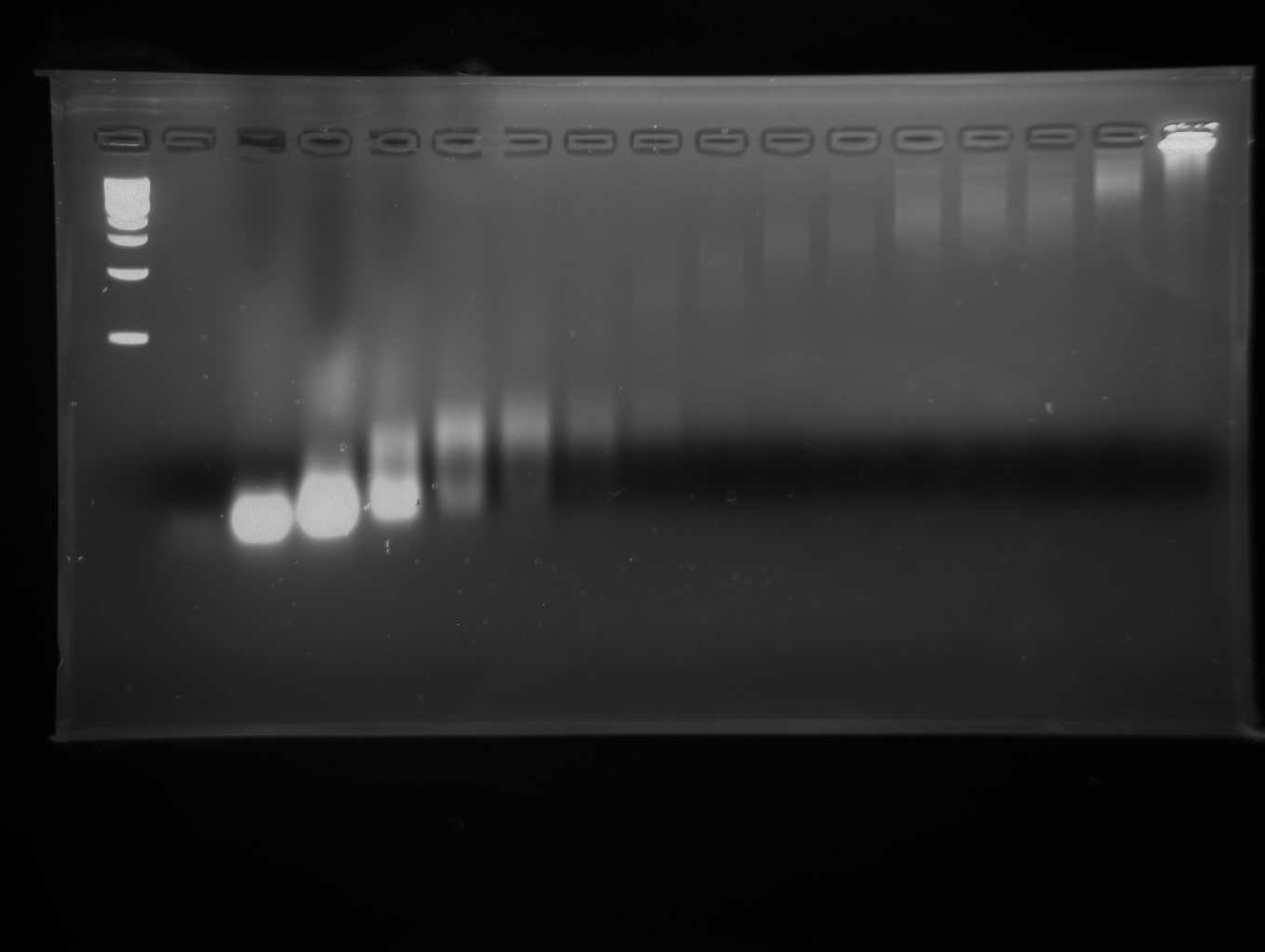

Supplement: Figure 10—source data 1. [file elife-108143-fig10-data1.zip › Figure 10-source data 1/OriginalgelFig10A.tiff]

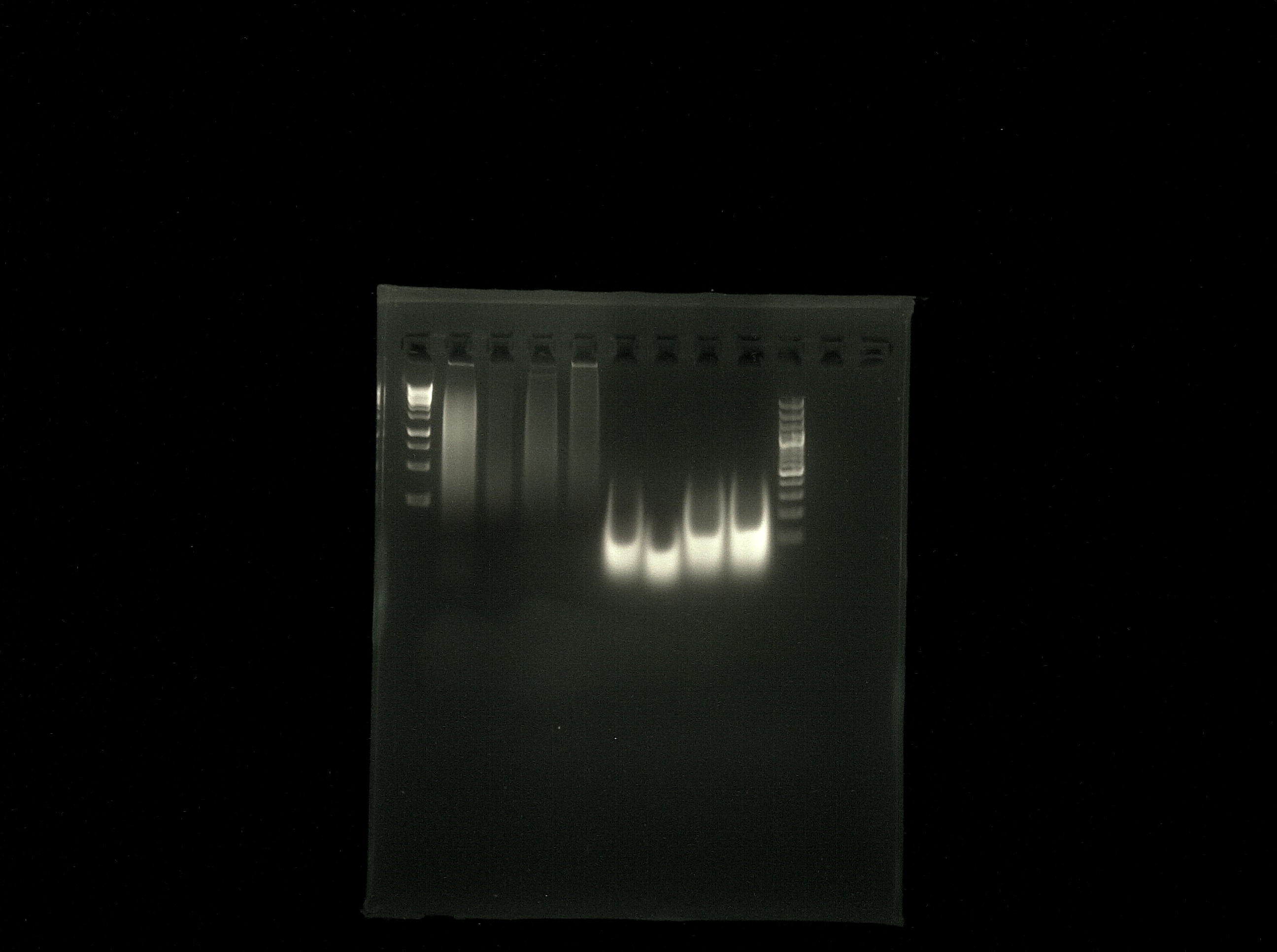

Supplement: Figure 10—source data 1. [file elife-108143-fig10-data1.zip › Figure 10-source data 1/originalgelFig10BC.TIF]

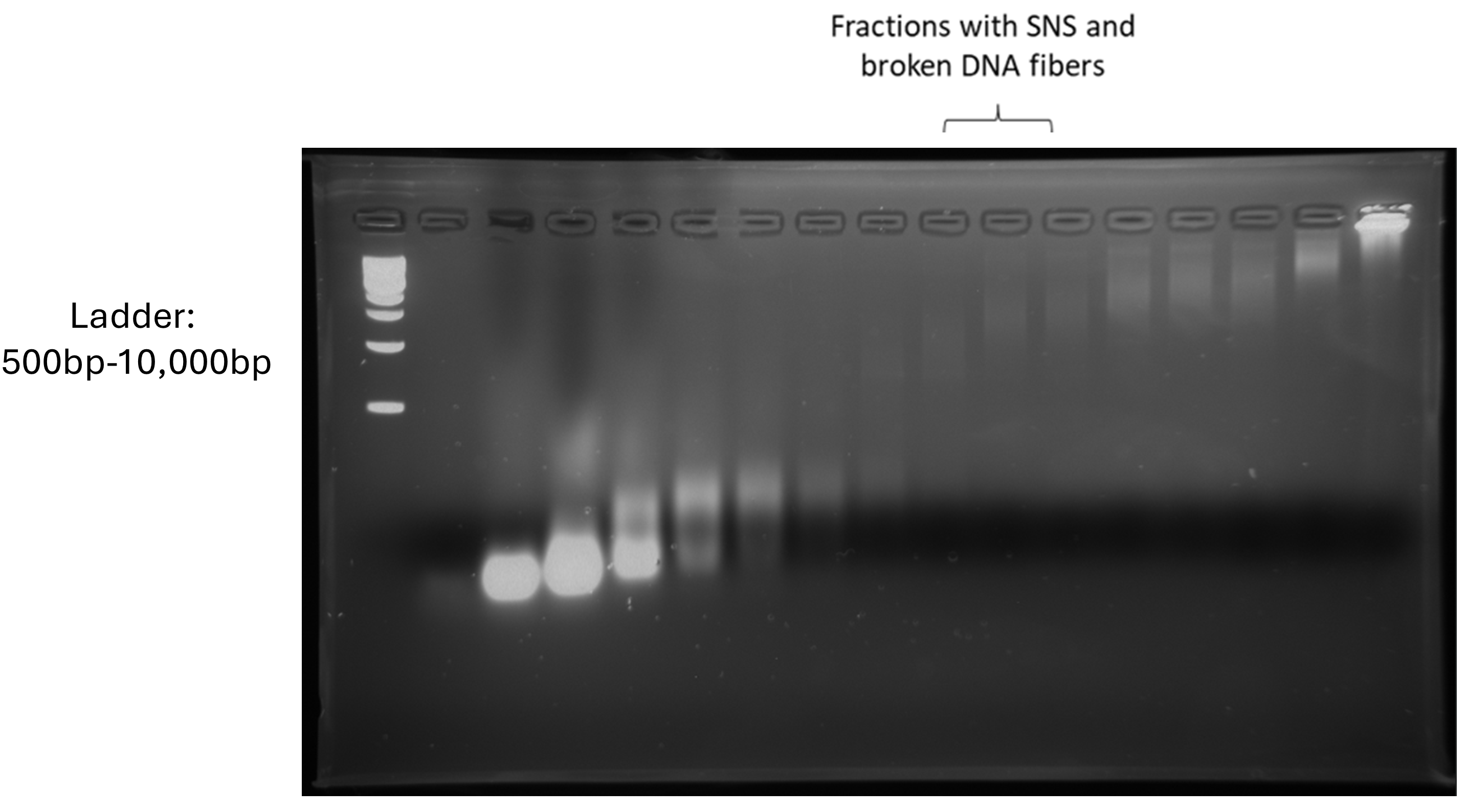

Supplement: Figure 10—source data 2. [file elife-108143-fig10-data2.zip › Figure 10-source data 2/AnnotateduncropgelFig10A.tif]

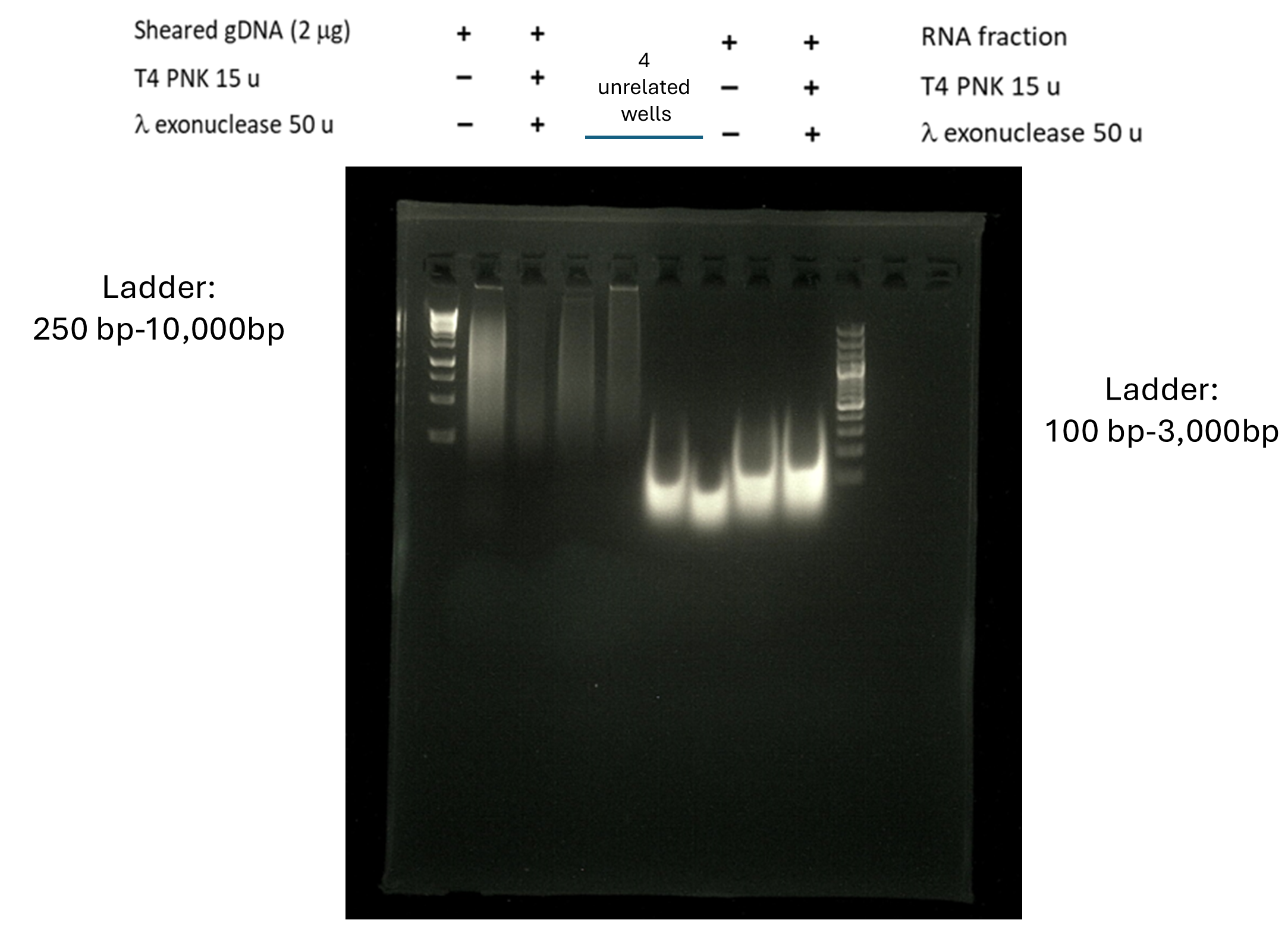

Supplement: Figure 10—source data 2. [file elife-108143-fig10-data2.zip › Figure 10-source data 2/AnnotateduncropgelFig10BC.tif]
