# Supplementary material for: Stranded short nascent strand sequencing reveals the topology of DNA replication origins in Trypanosoma brucei
Source: eLife. 2026 May 15;14:RP108143. doi: 10.7554/eLife.108143 (PMC13179062; doi:10.7554/eLife.108143)
Supplement: Supplementary file 1. — The numbers of reads obtained after paired-end sequencing, numbers of reads, peaks and mapped origins after different steps of bioinformatic analysis are presented for three biological replicates of PCF and BSF cells. Each replicate contained an SNS-enriched sample and SNS-depleted control. (+) and (-) peaks called present the number of peaks called and localised on the plus or minus DNA strand, respectively. Filtered peaks (+) and (-) represent pairs of peaks with divergent orientation (first (-) followed by (+) peak) (Methods). [file elife-108143-supp1.docx]

**Supplementary Table 1.** Numbers of reads

|  |  |  |  | **Peaks called against SNS depleted control** | | | |
| --- | --- | --- | --- | --- | --- | --- | --- |
|  | **Total reads** | **Reads after trimming and deduplication** | **Mapped reads** | **(+) peaks called** | **(-) peaks called** | **filtered peaks (+) and (-)** | **mapped ORIs** |
| PCF repl 1 SNS enriched | 35377234 | 26331533 | 1990221 | 890 | 919 | 76 | 38 |
| PCF repl 1 SNS depleted | 75161248 | 48009990 | 3310426 |  |  |  |  |
| PCF repl 2 SNS enriched | 40265002 | 29772101 | 2119887 | 2101 | 2660 | 170 | 85 |
| PCF repl 2 SNS depleted | 44506300 | 37022757 | 2724281 |  |  |  |  |
| PCF repl 3 SNS enriched | 67335770 | 57786492 | 4206187 | 5788 | 5381 | 904 | 452 |
| PCF repl 3 SNS depleted | 34346800 | 28214856 | 1999576 |  |  |  |  |
| BSF repl 1 SNS enriched | 32609118 | 21305027 | 1289981 | 2028 | 1866 | 462 | 231 |
| BSF repl 1 SNS depleted | 32673418 | 14195198 | 5432187 |  |  |  |  |
| BSF repl 2 SNS enriched | 49794683 | 43417511 | 2917335 | 7592 | 8268 | 1436 | 718 |
| BSF repl 2 SNS depleted | 42316922 | 36253185 | 2682328 |  |  |  |  |
| BSF repl 3 SNS enriched | 28145055 | 22793377 | 1548353 | 2986 | 3158 | 614 | 307 |
| BSF repl 3 SNS depleted | 28234323 | 21022409 | 1411179 |  |  |  |  |

The numbers of reads obtained after paired-end sequencing, numbers of reads, peaks and mapped origins after different steps of bioinformatic analysis are presented for three biological replicates of PCF and BSF cells. Each replicate contained an SNS-enriched sample and SNS-depleted control. (+) and (-) peaks called presents the number of peaks called and localized on the plus or minus DNA strand, respectively. Filtered peaks (+) and (-) represent pears of peaks with divergent orientation (first (-) followed by (+) peak) (Methods
