# Supplementary material for: Stranded short nascent strand sequencing reveals the topology of DNA replication origins in Trypanosoma brucei
Source: eLife. 2026 May 15;14:RP108143. doi: 10.7554/eLife.108143 (PMC13179062; doi:10.7554/eLife.108143)
Supplement: Supplementary file 2. — DNA replication parameters obtained by DNA combing in two cell types (PCF and BSF) of T. brucei. The estimated numbers of origins were obtained by dividing the genomic sequence length (50.081 Mb) by either the median or the mean IODs calculated on combed fibres. [file elife-108143-supp2.docx]

**Supplementary Table 2. DNA replication parameters obtained by DNA combing**

|  | **PCF** | **BSF** |  |
| --- | --- | --- | --- |
| **Inter-origin distance (kb)** | | |  |
| Number of values | 144 | 101 |  |
| Minimum | 23.26 | 37.2 |  |
| 25% Percentiles | 106.1 | 145.7 |  |
| Median | 152 | 212.8 |  |
| 75% Percentiles | 227.1 | 284.1 |  |
| Maximum | 597 | 584.9 |  |
| Mean | 179.4 | 219.4 |  |
| **Velocity of replication forks (kb/min)** | | |  |
| Number of values | 205 | 127 |  |
| Minimum | 0.6995 | 0.5525 |  |
| 25% Percentiles | 1.528 | 2.13 |  |
| Median | 1.724 | 2.408 |  |
| 75% Percentiles | 1.935 | 3.01 |  |
| Maximum | 3.212 | 4.053 |  |
| Mean | 1.754 | 2.56 |  |
| **Asymmetry of replication forks (long/short fork ratio)** | | | |
| Number of values | | 64 | 39 |
| Minimum | | 1 | 1 |
| 25% Percentiles | | 1.02 | 1.012 |
| Median | | 1.068 | 1.097 |
| 75% Percentiles | | 1.226 | 1.199 |
| Maximum | | 2.184 | 2.645 |
| Mean | | 1.163 | 1.189 |
| **Lengths of the analysed fibres (kb)** | | | |
| Number of values | | 187 | 151 |
| Minimum | | 104.3 | 171.4 |
| 25% Percentiles | | 330.1 | 361.2 |
| Median | | 446.9 | 446.9 |
| 75% Percentiles | | 516.9 | 511.6 |
| Maximum | | 1363 | 1146 |
| Mean | | 459.6 | 464.3 |
| **Estimated number of active origins per cell** | | | |
| Genomic sequence (50.081 Mb): median IOD | | 329 | 235 |
| Genomic sequence (50.081 Mb): mean IOD | | 279 | 228 |

**DNA replication parameters obtained by DNA combing in two cell types (PCF and BSF) of *T. brucei***. The estimated numbers of origins were obtained by dividing the genomic sequence length (50.081 Mb) by either the median or the mean IODs calculated on combed fibres.
